# Myoinositol in Autoimmune Thyroiditis

**DOI:** 10.3389/fendo.2022.930756

**Published:** 2022-06-28

**Authors:** Sabrina Rosaria Paparo, Silvia Martina Ferrari, Armando Patrizio, Giusy Elia, Francesca Ragusa, Chiara Botrini, Eugenia Balestri, Fabrizio Guarneri, Salvatore Benvenga, Alessandro Antonelli, Poupak Fallahi

**Affiliations:** ^1^ Department of Surgical, Medical and Molecular Pathology and Critical Area, University of Pisa, Pisa, Italy; ^2^ Department of Clinical and Experimental Medicine, University of Pisa, Pisa, Italy; ^3^ Department of Emergency Medicine, Azienda Ospedaliero-Universitaria Pisana, Pisa, Italy; ^4^ Department of Clinical and Experimental Medicine - Dermatology, University of Messina, Messina, Italy; ^5^ Department of Clinical and Experimental Medicine - Endocrinology, University of Messina, Messina, Italy; ^6^ Master Program on Childhood, Adolescent and Women’s Endocrine Health, University of Messina, Messina, Italy; ^7^ Interdepartmental Program of Molecular & Clinical Endocrinology and Women’s Endocrine Health, University Hospital Policlinico “G. Martino”, Messina, Italy; ^8^ Department of Translational Research and New Technologies in Medicine and Surgery, University of Pisa, Pisa, Italy

**Keywords:** myoinositol, autoimmune thyroiditis, hypothyroidism, CXCL10, chemokines

## Abstract

Myoinositol (Myo) is an isoform of inositol, a cyclic polyol with 6 hydroxyl groups. Myo is mainly derived from dietary intake while its endogenous production is generated from glucose by enzymatic reactions. Moreover, Myo is also synthesized *de novo* by catabolism of phosphatidylinositol (PI), phosphoinositides (PIP), and inositol phosphates (IP). Myo has a determinant role in thyroid function and autoimmune diseases as it regulates iodine organification and thyroid hormone biosynthesis by the formation of hydrogen peroxide (H_2_O_2_) in thyrocytes. Depletion of Myo that is involved in the thyroid stimulating hormone (TSH) signaling pathway, may cause the development of thyroid diseases such as hypothyroidism. TSH levels significantly decreased in patients with subclinical hypothyroidism, with or without autoimmune thyroiditis, after treatment with Myo plus Selenium (Myo+Se). In addition to TSH, antithyroid autoantibodies are reduced. This review summarizes the role of Myo in the thyroidal physiology and its role in the management of some thyroid diseases.

## Introduction

In the past few years, medical and public attention on nutraceuticals has grown. Nutraceuticals, also known as dietary supplements, are considered complementary medicines, defined as a “food, or parts of a food, that provide medical or health benefits, including the prevention and treatment of disease”. Presently, they are contemplated for the prevention of different pathological conditions, including chronic thyroid diseases and associated disorders. In fact, beyond iodine, which is necessary for thyroid physiology, other dietary components are involved in thyroid homeostasis and for this reason their clinical use has been questioned and evaluated. Among these, Inositol (Ins) is one of the most studied and prescribed.

With this manuscript, we aim to review Ins and its derivates knowledge, in the light of their role in thyroid physiology and pathology, and their potential clinical impact focusing on *in vitro* and *in vivo* studies reported in previous scientific literature (1).

## Myoinositol

Ins is a cyclic polyol with 6 hydroxyl groups and exists in 9 possible isoforms. Myoinositol (Myo) is the first isoform of Ins that has been described and the most commonly found (more than 99%) inside eukaryotic cells (2).

Myo is mainly derived from dietary intake: either as free form or as phytate (IP6). Vegetables are rich in IP6, while animal-derived foods are abundant of free Ins. The exogenous IP6 is converted by bacterial phytases in free Myo, orthophosphate, or Ins-phosphate metabolites (i.e., mono-, di-, tri-, tetra-, and penta-phosphate esters). Beans, citrus fruits (except lemons), nuts, and cereals have a high content of Ins (3).

Common Western diets account for an intake of about 1 g per day of Myo. Its gastro-intestinal absorption is guaranteed by two transporters [sodium/myo-inositol channels type 1 (SMIT1) and type 2 (SMIT2)], which are expressed on duodenum and jejunum mucosal cells (4).

In humans, Myo is also generated from glucose by enzymatic reactions: a) hexokinase convert glucose into glucose-6-phosphate (5), that is subsequently isomerized into inositol-3-phosphate (IP3) by an enzyme called D-3-myo-inositol-phosphate synthase (MIPS1, Ino1, or inositol synthase) (6). This enzymatic pathway up to 2 g Myo/day, are produced in each kidney, for a total of 4 g/day (5).

Moreover, Myo is also synthesized *de novo* by catabolism of phosphatidylinositol (PI), phosphoinositides (PIP), and inositol phosphates (IP) and afterward, the diacylglycerol-mediated reaction is used to build up new PIP (7). Finally, in mammalians, Myo is degraded in the kidney (8, 9).

To summarize, the human body pool of Myo is determined by three distinct pathways: 1) enteral absorption and renal clearance; 2) recycling between plasma, interstitial, and intracellular compartments; and 3) endogenous production and catabolism.

Cell membranes of all body tissues are made of phospholipids of which Ins is a constitutive element, and Myo is its most widespread isoform. Ins is involved in different physiological processes including in the neuronal transmission, shuttling of phospholipids, other fatty acid groups between cell membranes, intracellular effects of insulin, and intracellular calcium homeostasis. The central nervous system is the main Myo recipient, where it seems to promote emotional and mental wellness. Furthermore, in women, it exerts key functions in preserving ovarian wellbeing and glucose tolerance (10).

PIPs, IP, glycosylphosphatidylinositols (GPIs), IP3, inositol-phosphoglycans (IPGs), and PI derive from Myo-containing phospholipid and they play a role in the biochemical cascade which transmits a chemical signal through a cell as a series of molecular events called signal transduction (5, 11). In particular, numerous hormones such as thyroid stimulating hormone (TSH), luteinizing hormone (LH), follicle stimulating hormone (FSH), and insulin, transmit their function through the PI signal pathway where the phospholipase C (PLC) hydrolyzes phosphatidylinositol-4,5-biphosphate (PIP2) in two second messengers: IP3, and diacylglycerol (DAG), which in turn, open Ca^2+^ channels of the smooth endoplasmic reticulum and mitochondria membranes and induce protein kinase C (PKC), with subsequent cellular responses (12).

As a result, Myo homeostasis impairment could potentially affect several physiological cellular mechanisms that may translate to a broad range of disorders, ranging from thyroid diseases, fertility impairment, polycystic ovary syndrome (PCOS), neurological diseases, and diabetes (13).

Thyroid hormones (TH) homeostasis is controlled through both the PLC-dependent inositol phosphate Ca^2+^/DAG and the cyclic AMP (cAMP) cascade (14), both activated by the TSH and its receptor (TSHR) binding. The cAMP cascade regulates thyrocytes development and differentiation and TH secretion (15), while the PLC-dependent inositol phosphate Ca^2+^/DAG pathway results in enhanced H_2_O_2_ production, which is needed for iodine incorporation and TH synthesis (16, 17). Therefore, Myo and its derivates are essential in thyroid physiology, as demonstrated *in vitro*, by active accumulation of Myo and by inositol phosphate formation in thyrocytes under increased TSH level (18, 19). Moreover, metabolomic studies indicate that hypothyroid patients require higher Myo level than healthy subjects (20), suggesting that Myo may limit thyroid functions impairment by increasing iodine availability for thyrocytes (21).

## Autoimmune Thyroid Diseases (AITD)

Autoimmune thyroid diseases (AITD) include the chronic autoimmune thyroiditis [Hashimoto’s thyroiditis (HT)], which is the most common cause of hypothyroidism in iodine-sufficient areas, along with Grave’s disease (GD), a syndrome that consists in hyperthyroidism, goiter, thyroid eye disease, and occasionally pretibial dermopathy. Both of these disorders are considered the result of the combination of genetic susceptibility with environmental factors and they are characterized by the presence of high serum levels of autoantibodies against one or more thyroid antigens, along with a diffuse lymphocytic infiltration of the thyroid tissue, which includes predominantly thyroid-specific T cells and especially, in HT, and B cells (22, 23). However, AITD can be present even in the absence of circulating antithyroid autoantibodies (24).

Women have a higher risk of developing AITD than men (25). The prevalence of AITD is higher in iodine-sufficient areas (25) and where there is an increased iodine intake (26).

AITD are very common in the general population and it shows an increased association with other autoimmune diseases, particularly with connective tissue disease [i.e., systemic sclerosis (SSc), Sjogren syndrome (SS), vitiligo, systemic lupus erythematosus (SLE), rheumatoid arthritis (RA), and sarcoidosis] (27–29).

Genetic predisposition for AITD includes the familial clustering of the disease, a concordance rate in monozygotic twins (20-40%), and a sibling risk ratio of approximately 17 (30).

Approximately 70% of the genes associated with the risk of AITD take part in T cell functions, underlying the role of these cells in AITD pathogenesis (31).

AITD occurrence is associated with environmental factors in about 20% of cases. The external insult causes a cellular/tissue damage that switches on the innate immune system leading to the progression of an AITD in the presence of a genetic susceptibility (27, 32).

Radiation exposure, infection, sex steroids, stress, pregnancy, and iodine intake are the known possible precipitating environmental factors for AITD. Fetal microchimerism within the maternal thyroid is also a possibility (26). Smoking is a risk factor for Graves’ hyperthyroidism and even a stronger risk factor (33, 34) for Graves’ Ophthalmopathy.

The thyroidal tissue expresses specific selenoproteins and the lack of selenium is involved in the onset of thyroid autoimmunity, whereas its supplementation protects from AITD (35).

Among infections, people with hepatitis C virus (HCV) infection showed (36–38) higher prevalence of AITD thyroid autoantibodies levels.

The immune cells infiltrate the AITD thyroid and it includes CD4+ and CD8+ T cells, CD19+ B cells, macrophages, and plasma cells. B lymphocytes also act as antigen presenting cells (APCs), activating naïve autoreactive CD4+ T cells by presenting thyroid autoantigens (Tg and TPO) making the gland the major site of thyroid antibody secretion against Tg and TPO antigen (39).

## Subclinical Hypothyroidism

The PLC-dependent inositol phosphate Ca^2+^/DAG pathway regulates the biosynthesis of H_2_O_2_ which is needed for iodine organification and TH biosynthesis making Myo-containing phospholipids derivates (IP3, PI, PIP, IPGs and GPIs) impairment an element of disruption for thyroid physiology with subsequent potential development of hypothyroidism (40, 41). Recently, several studies have explored the possible role of Myo in the management of subclinical hypothyroidism (SCH) associated with AITDs (SCH-AITD). In 2013, the authors of another study, in order to evaluate oral Myo in women with SCH-AITD, uniformly randomized 48 women with TSH values with the interval of 4.01-9.99 mIU/L and with antithyroglobulin (AbTg), and antithyroid peroxidase (AbTPO) positivity in two harms: in one group, 600 mg of Myo plus 83 mcg of selenium (Myo+Se) were administrated for 6 months, while in the other, only 83 mg of selenium (Se) was administered the same period of time. At the end of the study, the Myo+Se group showed 31% of TSH drop and a 44% and 48% reduction of AbTPO and AbTg, respectively (p<0.01), while the Se group demonstrated only lower antibodies with no significant variation in the TSH level (42).

Later, 86 patients (men and women) suffering from SCH and HT, were managed with Myo+Se, showing significant reduction in TSH values (p ≤ 0.001) after 6 months of therapy and a clear amelioration in their quality of life, after being assessed with a subjective examination form (43). In 2017, another study evaluated 168 patients affected by HT with TSH within the 3 and 6 mIU/mL range, dividing them in two groups in which Myo+Se, or only Se (83 μg) were prescribed. After 6 months, the researchers noticed a compelling recovery in the thyroid functions test with Myo+Se therapy (44). Following data confirmed these findings (45), even in a different clinical setting. In fact, in 2018, the efficacy and the safety of Myo+Se supplementation was examined in pregnant women with TSH levels laying between 1.6-2.5 μIU/ml (600 mg Myo plus 83 μg Se, daily throughout pregnancy) observing more patients with normal TH in the treated group than in the control group (94.1% *vs* 68.7%) (46). Moreover, Morgante et al. reported that after 6 months, in insulin resistant PCOS patients on Ins+metformin therapy *vs.* metformin alone, TSH dropped significantly (p<0.05) in the Ins-combined treatment group (47).

There are further results supporting beneficial Myo impact on patients with SCH and HT in a time-dependent manner with TSH declined, over a treatment period of three months, by 21% (48) and even more steadily when the administration is prolonged for a 1 year (40).

## Myoinositol and CXCL10

Previous studies showing antithyroid autoantibodies levels decline together with reduction in TSH values (42, 45), and hypothesized an immune-modulatory effect originated by Myo-based therapy. This has been further supported by the measurement, before and after treatment, of C-X-C motif chemokine ligand 10 (CXCL10) levels. The CXCL10, also known as IFN-γ-inducible protein 10 (IP-10), through its receptor [chemokine (C-X-C motif) receptor 3 (CXCR3)], is implicated in the immune-pathogenesis of numerous autoimmune diseases, (i.e., GD and orbitopathy, type 1 diabetes, mixed cryoglobulinemia, SLE, SS, or SSc) (49, 50).

Beyond CD4+, CD8+, and natural killer (NK), it has also been demonstrated that thyrocytes are able to release CXCL10 under the stimulant effect of IFN-γ. Indeed, AITD patients, especially if complicated by hypothyroidism and ultrasonographic hypoechogenicity of the gland (sign of a more lymphomonocytic infiltration), show high serum CXCL10. Hence, CXCL10 in peripheral fluids could be considered a marker of T helper (Th)1 type immunity, whose circulating levels directly correlate with the intensity and magnitude of thyroid auto-inflammatory state (45).

In 2017 Ferrari SM et al., studied the effect of the association of Myo and Se (600 mg/83 mcg), given twice per day for a 6-month period, on 21 patients with newly diagnosed autoimmune thyroiditis (AT). After the treatment period, besides the significant reduction in TSH and AbTg levels, they also reported a decline, even if not statistically significant, of CXCL10 levels compared to initial values (114 ± 46, vs. 144 ± 54, pg/mL, respectively; p=0.061) (45).

The exact mechanisms through which Myo and Se can influence the immune-response are still unknown, demanding further investigations. However, preliminary *in vitro* studies performed on blood mononuclear cells (PBMC), taken from either HT and normal controls and subjected to H_2_O_2_-induced oxidative stress, revealed that Myo+Se reduced the burden of several cytokines, including CXCL10, CCL2, CXCL9, and the H_2_O_2_-mediated genotoxicity (51–53).

Finally, Myo deficiency has also been associated with an increased thyroid cancer risk. In fact, preliminary metabolomic studies which examined samples of normal thyroids in comparison with those of glands carrying benign nodular diseases, follicular adenoma, and thyroid carcinoma reported lower Myo thyroid tissue amount with malignancy (54). Conversely, in 2018, a retrospective investigation examined the effects, after 6 months, of 600 mg Myo plus 83 mcg Se supplementation on benign thyroid nodules [class I and II defined by AACE/ACE/AME Guidelines (55)] in patients with SCH. Observations were a reduction of the size (16.72 ± 1.32 vs 12.44 ± 1.81), number (1.39 ± 0.16 vs 1.05 ± 0.15), and elasticity score (1.80 ± 0.13 *vs* 1.24 ± 0.18) of thyroid nodules (56).

## Conclusions

Former *in vitro* and *in vivo* studies revealed a potential favorable impact of Myo supplementation on subclinical hypothyroidism and autoimmune thyroiditis, emphasizing the crucial role of Myo in the homeostasis of the endocrine system, including the thyroid and other organs. In fact, as a source of second messengers, such as IP3, Myo is involved in the TH biosynthesis and metabolism and thyrocytes need physiological levels of Myo to ensure the euthyroid status. Moreover, reduced levels of thyroid antibodies, pro-inflammatory chemokines (i.e., CXCL10), and oxidative stress observed after Myo employment advocate for the immune-modulatory effect of the compound that could be clinically relevant to prevent euthyroid AT and SCH patients to develop overt thyroid dysfunctions. While these results need to be confirmed by larger studies and clinical trials, and also to further elucidate the biochemical mechanisms, Myo treatment turns out to be a compelling approach on the management of subclinical AT and hypothyroidism.

## Author Contributions

SRP, SMF, AP, FG, SB, AA, and PF conceived the paper. All authors reviewed and approved the final version of the manuscript.

## Funding

The cost of the publication has been sustained by LoLi Pharma.

## Conflict of Interest

The authors declare that the research was conducted in the absence of any commercial or financial relationships that could be construed as a potential conflict of interest.

## Publisher’s Note

All claims expressed in this article are solely those of the authors and do not necessarily represent those of their affiliated organizations, or those of the publisher, the editors and the reviewers. Any product that may be evaluated in this article, or claim that may be made by its manufacturer, is not guaranteed or endorsed by the publisher.
